# Analysis of the distribution characteristics and influencing factors of viruses carried by atmospheric PM_2.5_ based on metaviromics

**DOI:** 10.3389/fpubh.2025.1616737

**Published:** 2025-06-25

**Authors:** Wenli Wang, Yongxin Wang, Haoneng Hu, Quan Zhou, Shuling Kang, Yu Jiang, Jianjun Xiang, Jing Wu, Jing Li, Zhiwei Chen, Chuancheng Wu

**Affiliations:** ^1^School of Public Health, Fujian Medical University, Fuzhou, China; ^2^Fuzhou Center for Disease Control and Prevention, Fuzhou, China

**Keywords:** gene sequencing, viruses, PM_2.5_, metaviromics, community structure, environmental factors, abundance

## Abstract

**Background:**

Fine particulate matter (PM_2.5_) is a well-known air pollutant and has been suggested as a potential vector for airborne viruses, raising public health concerns. This study employed metaviromic sequencing to systematically analyze the composition, temporal–spatial distribution, and environmental influencing factors of viral communities in PM_2.5_ samples collected from Fuzhou, China, to identify potential high-risk viruses and the key factors influencing their presence.

**Methods:**

Three outdoor PM_2.5_ sampling sites were established in the city center, rural–urban fringe, and rural areas of Fuzhou. Samples were collected from December 2022 to August 2023. The collected PM_2.5_ samples underwent high-throughput sequencing and viral annotation, and statistical analysis along with multivariate regression analyses were used to investigate the characteristics of viral distribution and its influencing factors.

**Results:**

A total of 117 PM_2.5_ samples were collected. The viral community diversity in PM_2.5_ exhibited significant seasonal variation (*p* < 0.05), with the highest number of viral species detected in winter at both the genus and species levels. In terms of regional distribution, the highest number of viruses was found in city center and the lowest in rural areas, while there were slight differences in viral composition among regions, these were not statistically significant. Additionally, analysis of environmental factors revealed that sulfur dioxide (SO_2_) in the air quality factor and wind speed in the meteorological factor influenced the relative abundance of viruses.

**Discussion:**

Urbanization and human activities may affect regional viral patterns, but the overall improved air quality in Fuzhou could have reduced regional disparities. Environmental factors such as SO_2_ and wind speed may influence viral survival and dispersion, suggesting that non-traditional pollutants warrant closer attention in the context of airborne virus transmission.

## 1 Introduction

Atmospheric particulate matter can remain suspended in the air for extended periods and adsorb various viral pathogens, making it a potential medium for their transmission and leading to long-range spread events (1–3). Compared with other atmospheric particles, PM_2.5_ has smaller particle sizes and slower settling velocities, allowing it to stay in the atmosphere for longer periods and spread over wider areas (4, 5). For example, in some industrial cities, PM_2.5_ can be transported to surrounding areas by wind, with transmission distances reaching up to hundreds of kilometers (6). Moreover, PM_2.5_ not only enhances the impact of viruses on the respiratory system and prolongs their infectivity, but its harmful chemical components may also damage lung function, creating a synergistic toxic effect with the viruses (7, 8). Research has shown that harmful constituents of PM_2.5_ can damage the mucosal barrier of the respiratory tract, enhancing viral invasion capacity (9), and are closely associated with elevated rates of respiratory illness and mortality. Therefore, PM_2.5_ can be considered an “ideal carrier” for viruses to facilitate short-distance or long-distance transmission.

Although existing studies have focused on the detection of airborne viruses, most of them still concentrate on specific pathogens or rely on targeted detection methods such as quantitative polymerase chain reaction, lacking a systematic study on the diversity and composition of airborne viruses. Metaviromics, as a cutting-edge approach in environmental virology, enables the extraction of total viral nucleic acids from samples followed by high-throughput sequencing. Combined with bioinformatics analysis, this technique allows for the identification and annotation of both known and potential viruses, thereby revealing the composition and dynamic characteristics of viral communities (10, 11). In recent years, it has been widely applied to the analysis of viruses in environmental matrices such as soil and water (12).

In this study, we employed metaviromic methods to systematically analyze the viral composition, dominant species, seasonal variation, and environmental factors associated with the abundance of human-host viruses in PM_2.5_ samples collected from different regions of Fuzhou, China. The findings aim to provide a scientific basis for atmospheric virology research and the health risk assessment of airborne pathogens. Furthermore, PM_2.5_ may serve as a valuable supplementary medium for future public health surveillance and early warning of viral outbreaks.

## 2 Methods

### 2.1 PM_2.5_ sampling sites

A total of three PM_2.5_ sampling sites were selected for this study: Qunzhong Road Primary School located in the center of Fuzhou City, Hongtang Central Primary School in the rural–urban fringe, and Shangjie Campus of Fujian Medical University in the rural. No major pollution sources were found near any of the sampling points. The sampling areas are shown in Supplementary Figure S1. Since 2016, Qunzhong Road Primary School and Hongtang Central Primary School have been established as PM_2.5_ monitoring and component analysis sites in Fuzhou.

### 2.2 PM_2.5_ sample collection

PM_2.5_ samples were collected every 3 days from December 10, 2022, to August 31, 2023, using the MH1205 constant-temperature, constant-flow air/particulate sampler. Quartz filter membranes were employed to collect PM_2.5_. All samples were collected for 72 h, with ambient air drawn in at an average flow rate of 100 L/min. The sampler is resistant to extreme weather and can carry out normal sampling work under adverse weather conditions, ensuring that the sampling process is not interrupted by the weather. The field deployment configuration of the MH1205 samplers is shown in Supplementary Figure S2 and the basic principle is shown in Supplementary Figure S3.

Before sampling, the filter membranes were baked in a muffle furnace at 400°C for 5 h to remove organic matter and enhance filter toughness. After baking, the filter membranes were stored in sterile filter boxes and equilibrated in a refrigerator at 4°C with low humidity for at least 24 h before initial weighing. All sampling instruments and materials were disinfected with 75% alcohol before use.

After sampling, the filter membrane was removed using sterilized, sterile tweezers and placed in a refrigerator to equilibrate for at least 24 h before final weighing. The membranes were then stored in a − 80°C freezer for subsequent analysis. Both the weighing and sealing processes were conducted within a sterile workbench. One blank membrane was collected once a month as a control.

### 2.3 Data collection

Air pollutant data, including PM_2.5_, PM_10_, SO_2_, NO_2_, O_3_, and CO indicators, and meteorological data, such as air pressure, air temperature, humidity, wind speed, and hours of sunshine, were collected for Fuzhou City from December 2022 to December 2023, All the data were obtained from the Fuzhou Environmental Protection Bureau of Fujian Province and Fuzhou Meteorological Bureau.

### 2.4 Construction of sequencing library and high-throughput sequencing

**Figure 1:**
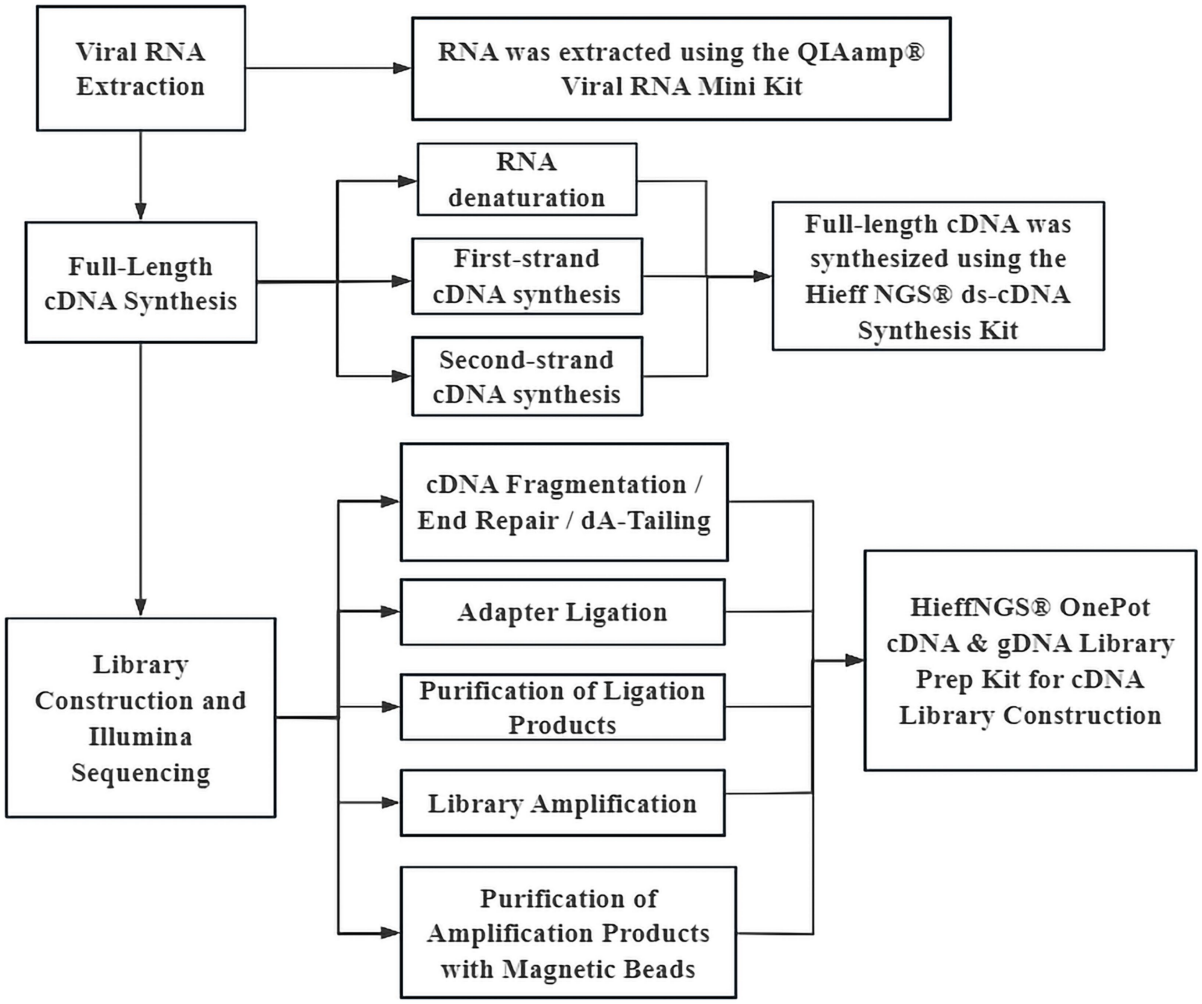
Flowchart of sequencing library construction.

Viral RNA was extracted from the collected PM_2.5_ samples to construct sequencing libraries (as shown in Figure 1). the quality was checked by quantitative detection and length distribution detection, The library samples were mixed according to a certain total amount of the samples, followed by double-end sequencing using Illumina’s NovaSeq 6,000 sequencing system PE150 platform. Finally, the sequencing data were downloaded remotely via an FTP server, and the data were verified using MD5 to ensure the integrity of data transmission.

### 2.5 Sequencing data analysis

Firstly, the raw sequencing reads were processed using the Mars-AS pathogen identification system developed by MicroFuture Bioinformatics Technology Co., Ltd., China. Quality control filtration, host sequence removal, and splicing assembly were performed to obtain taxonomic annotations of viral sequences. Based on the above data, the species information, compositional differences, and distributional characteristics among the samples were mined.

### 2.6 Statistical analysis

In this study, Microsoft Excel was used to clean and organize viral species-level annotation data and to establish a corresponding database. Based on the annotation results, the viral host information was retrieved using the taxonomy resources available in the online database of the National Center for Biotechnology Information (NCBI)^1^, and the viruses were classified into plant viruses, human pathogenic viruses, other animal viruses, and other viruses (including bacterial viruses, fungal viruses, etc.). SPSS 26.0 software was used to analyze the seasonal and regional distribution characteristics of viral communities and to perform intergroup comparisons. Multivariate regression analysis was conducted to identify factors influencing viral abundance. Venn diagrams illustrating interactions among viral groups were generated using R software version 4.3.2, and Origin 2022 software was used to rank and visualize the top 10 viral species by relative abundance.

## 3 Results

### 3.1 Basic data characteristics

#### 3.1.1 PM_2.5_ sample collection situation

The sample collection period of this study was from December 10, 2022, to August 31, 2023, covering the winter, spring, and summer seasons. A total of three sampling sites were established in the city center, rural–urban fringe, and rural areas of Fuzhou City, with 39 PM_2.5_ samples collected at each site, resulting in a total of 117 PM_2.5_ environmental samples. The mass weight distribution of the PM_2.5_ samples collected at each site across different seasons is provided in Supplementary Table S1.

#### 3.1.2 Quality control results of sequencing data

To ensure the accuracy of subsequent analyses, the raw sequencing data were subjected to strict quality control in this study. In next-generation sequencing, each base is assigned a quality score to indicate the accuracy of base calling. Q30 indicates a base quality value of 30, which equates to a sequencing error rate of 0.1%, or a base call accuracy of 99.9%. In this study, all 117 PM_2.5_ samples achieved Q30 scores above 85%, indicating high base-calling accuracy and low error rates.

Ultimately, the clean reads of high-quality sequences obtained in each season were 2,125,955,554 in winter, 1,480,030,864 in spring, and 1,123,681,932 in summer. The number of clean reads obtained from Qunzhong Road Primary School, Hongtang Central Primary School, and Fujian Medical University were 1,775,484,318, 1,570,584,154, and 1,383,599,878, respectively.

### 3.2 Seasonal distribution characteristics of the virus community

#### 3.2.1 Virus community species-genus level results

At the genus level, analysis of the viral genera detected in the PM_2.5_ samples showed that the winter group had the highest average number of viral genera, with a mean of 38.25 ± 13.160. The number of viral genera in individual winter samples ranged from 15 to 67. while it was lowest in the summer. The difference in the number of viruses at the genus level between seasons was analyzed by the rank-sum test and was statistically significant (*H* = 11.994; *p* < 0.02) (shown in Table 1).

**Table 1:** Genus-level statistics of virus communities in different seasons.

Season	Mean ± SD	P_25_	P_50_	P_75_	*H*	*p*
Winter	38.25 ± 13.160	29.0	37.0	48.0		
Spring	35.24 ± 12.500	26.5	35.0	43.5	11.994	0.02
Summer	28.39 ± 17.565	20.0	25.0	31.0		
Total	35.26 ± 14.264	25.0	34.0	44.0		

Similarly, at the species level, a statistical analysis of the number of viral species detected in the PM_2.5_ samples revealed that the highest number was observed in winter, while the lowest was in summer. The rank-sum test indicated that the differences in viral species counts across seasons were also statistically significant (*H* = 11.353; *p* < 0.03) (see Table 2).

**Table 2:** Species-level statistics of viral communities in different seasons.

Season	Mean ± SD	P_25_	P_50_	P_75_	*H*	*p*
Winter	40.60 ± 14.160	30.0	39.0	50.5		
Spring	36.32 ± 12.758	28.0	36.0	44.5	11.353	0.03
Summer	30.57 ± 17.581	20.0	18.0	35.0		
Total	37.13 ± 14.796	26.5	36.0	46.0		

#### 3.2.2 Analysis of viral community structure at the genus and species levels

**Figure 2:**
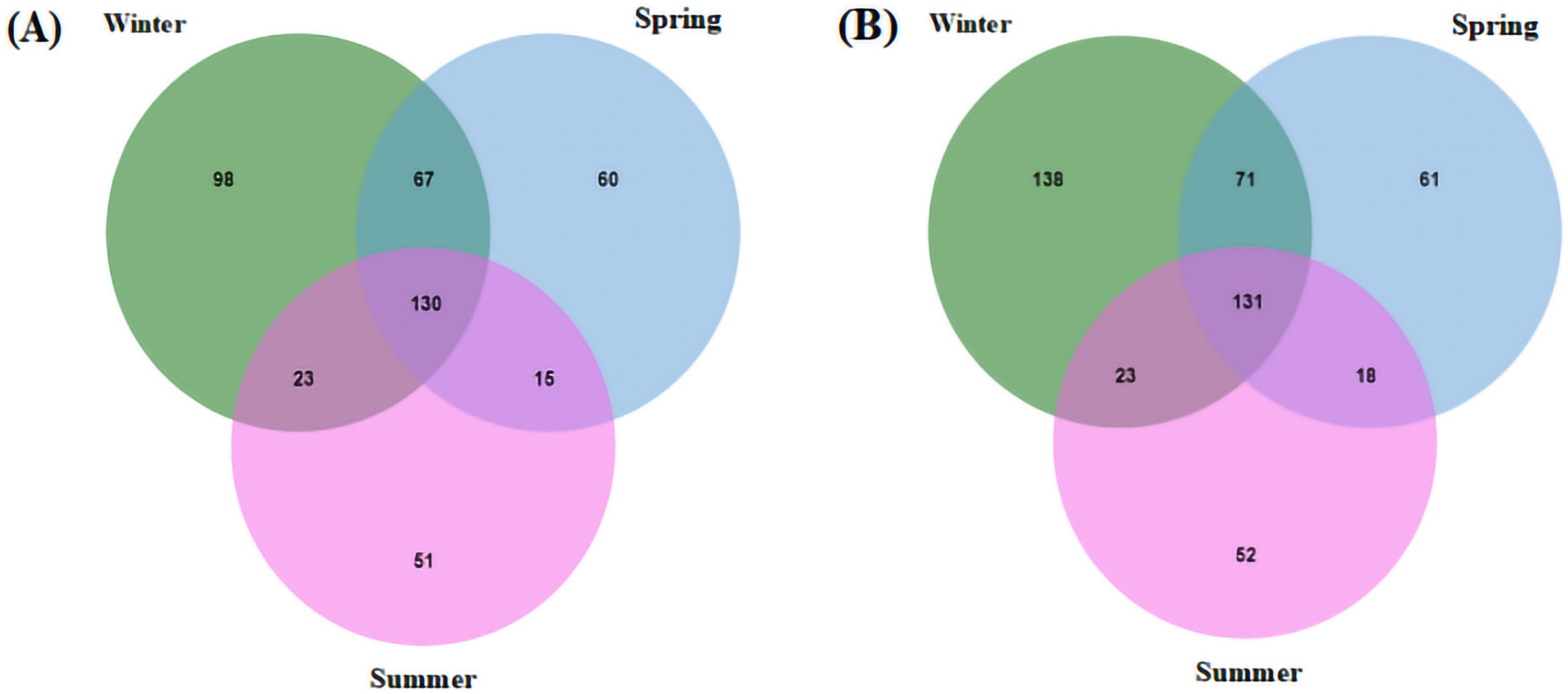
Seasonal interaction Venn diagram of virus community structure levels. **(A)** Genus level; **(B)** species level.

The seasonal interaction Venn diagram of the viral community in PM_2.5_ at the genus level is shown in Figure 2. A total of 130 viral genera and 131 viral species were shared among all three seasons.

Cross-seasonal analysis between winter and spring revealed 197 shared viral genera. Notably, the Cavally virus, belonging to the *Alphamesonivirus 1* genus, was detected only in winter and not in spring. In addition, the presence of *Beta-papillomavirus type 2* showed significant seasonal variation between these two seasons. Interestingly, several viral species within the *Enterovirus B* genus were detected in winter but not in spring.

Comparisons between spring and summer, as well as winter and summer, showed similar patterns, with two viral genera exhibiting clear seasonal variation at the species level. For example, bovine coronavirus, a member of the *Betacoronavirus 1* genus, was detected only in winter and spring but not in summer, whereas the human coronavirus OC43, from the same genus, was consistently detected across all three seasons. The statistics of viruses uniquely detected in spring are provided in Supplementary Table S2.

#### 3.2.3 Seasonal distribution characteristics of virus classifications

Based on host types, the viruses identified at the species level were categorized into plant viruses, human host viruses, other animal viruses, and other viruses. According to their genetic material, they were further classified as either DNA viruses or RNA viruses. A comparative analysis of these viral categories across winter, spring, and summer revealed that the detection numbers of plant viruses, human host viruses, and other animal viruses were highest in winter, followed by spring, and lowest in summer. The seasonal differences in viral composition among different host types were statistically significant (*χ^2^* = 43.180; *p* < 0.001) (Table 3).

**Table 3:** Statistics on seasonal distribution of virus classifications (%).

Classification	Season			Total	*χ^2^*	*p*
	Winter	Spring	Summer			
Plant viruses	56.7	29.6	13.7	100.0		
Human host viruses	47.9	31.7	20.3	100.0	43.180	0.000
Other animal viruses	46.7	36.5	16.8	100.0		
Other viruses	46.3	37.0	16.7	100.0		

#### 3.2.4 Composition of viral community structure

**Figure 3:**
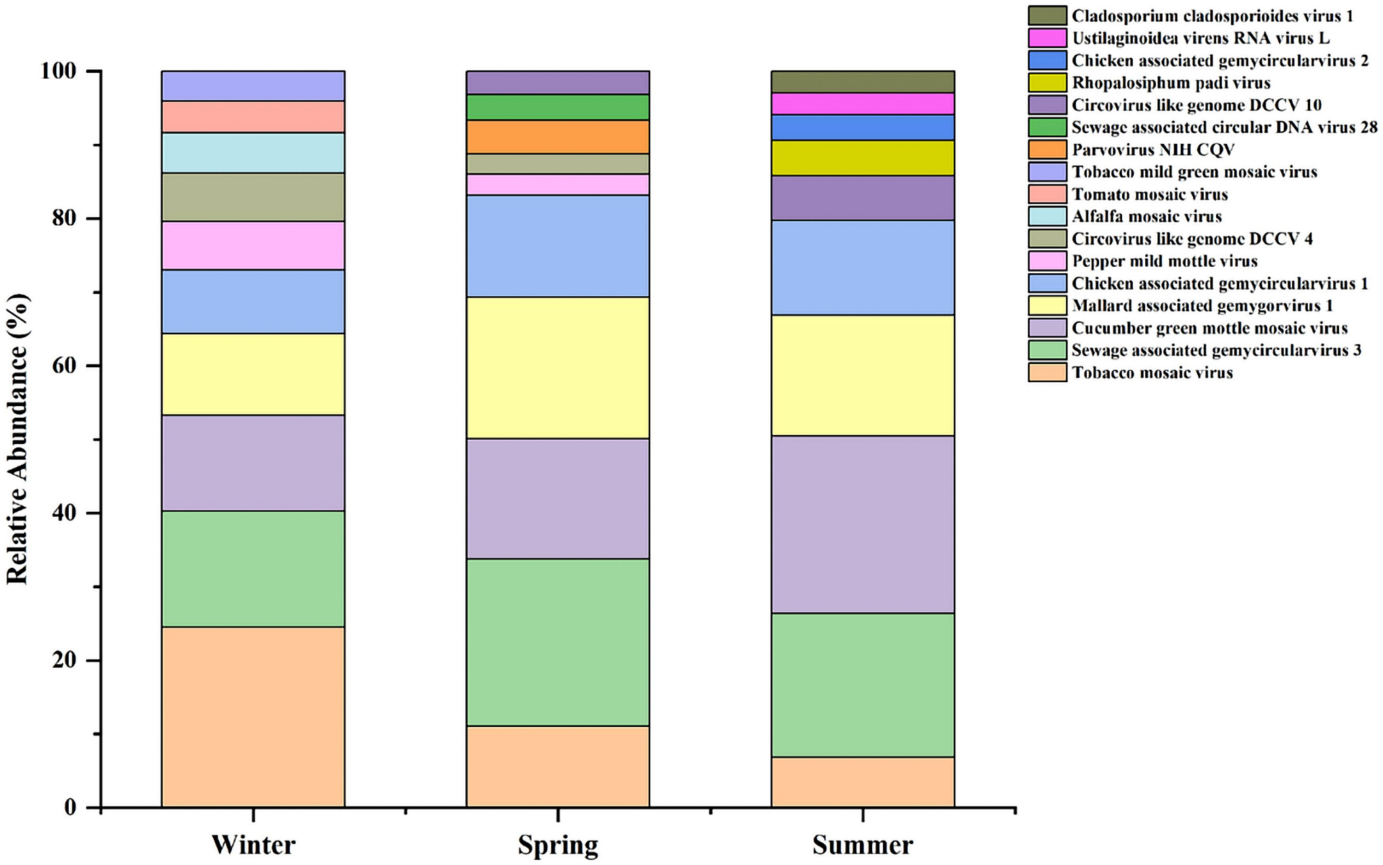
Seasonal distribution of the top 10 most abundant viral species.

Comparative analysis of the relative abundance of viruses in winter, spring, and summer revealed the presence of dominant viral genera and species in each season, with significant differences (see Figure 3). Notably, the dominant viral genus and species in spring and summer were consistent, whereas in winter, the dominant genus and species differed, suggesting that the viral community structure in winter may be more complex.

Overall, there were significant differences in the dominant virus species between seasons, and the relative abundance levels varied widely. Overall relative abundance of viruses was highest in winter, followed by spring, and lowest in summer, indicating a relatively high viral load in winter.

Further analysis of the top 10 most abundant viruses across the three seasons revealed a high degree of overlap (Supplementary Table S4). At the species level, 5 types of viruses are common to all three seasons. Additionally, genus-level analysis showed that human host-associated viruses were detected in all seasons; however, they belonged to different viral genera, indicating seasonal variations in the composition of the human-related viral community.

### 3.3 Regional distribution characteristics of viral communities

#### 3.3.1 Statistical analysis of viral genera and species level

Statistical analysis of the viruses in the PM_2.5_ samples showed that at the genus level, the city center group had the highest number and widest range of viral genera, with individual samples containing between 9 and 95 genera. By contrast, the rural group had the lowest number of viral genera. However, ANOVA revealed that the differences in the number of viral genera across the regions were not statistically significant (*F* = 1.461; *p* = 0.236) (Table 4).

**Table 4:** Genus-level statistics of virus communities in different regions.

Site	Mean ± SD	P_25_	P_50_	P_75_	*F*	*p*
City center	38.32 ± 16.848	29.0	37.0	48.0		
Rural–urban fringe	34.00 ± 11.427	26.5	35.0	43.5	1.461	0.236
Rural	33.27 ± 13.696	20.0	25.0	31.0		
Total	35.28 ± 14.282	25.0	34.0	44.0		

A similar trend was observed at the species level: the city center group had the highest number of annotated viral species, followed by the rural–urban fringe, and the rural group had the lowest. Again, ANOVA showed no statistically significant differences in viral species counts among the regions (*F* = 1.434; *p* = 0.243) (Table 5). Notably, although there were gradient differences in group means, the large standard deviations and wide data ranges suggest significant heterogeneity in viral diversity within the sample.

**Table 5:** Species-level statistics of virus communities across different regions.

Site	Mean ± SD	P_25_	P_50_	P_75_	*F*	*P*
City Center	40.07 ± 16.999	29.5	38.0	50.5		
Rural–urban fringe	36.51 ± 13.117	25.0	35.0	46.0	1.434	0.243
Rural	34.51 ± 13.617	26.5	34.0	41.0		
Total	37.13 ± 14.264	26.5	36.0	46.0		

#### 3.3.2 Regional structure analysis of viral communities

**Figure 4:**
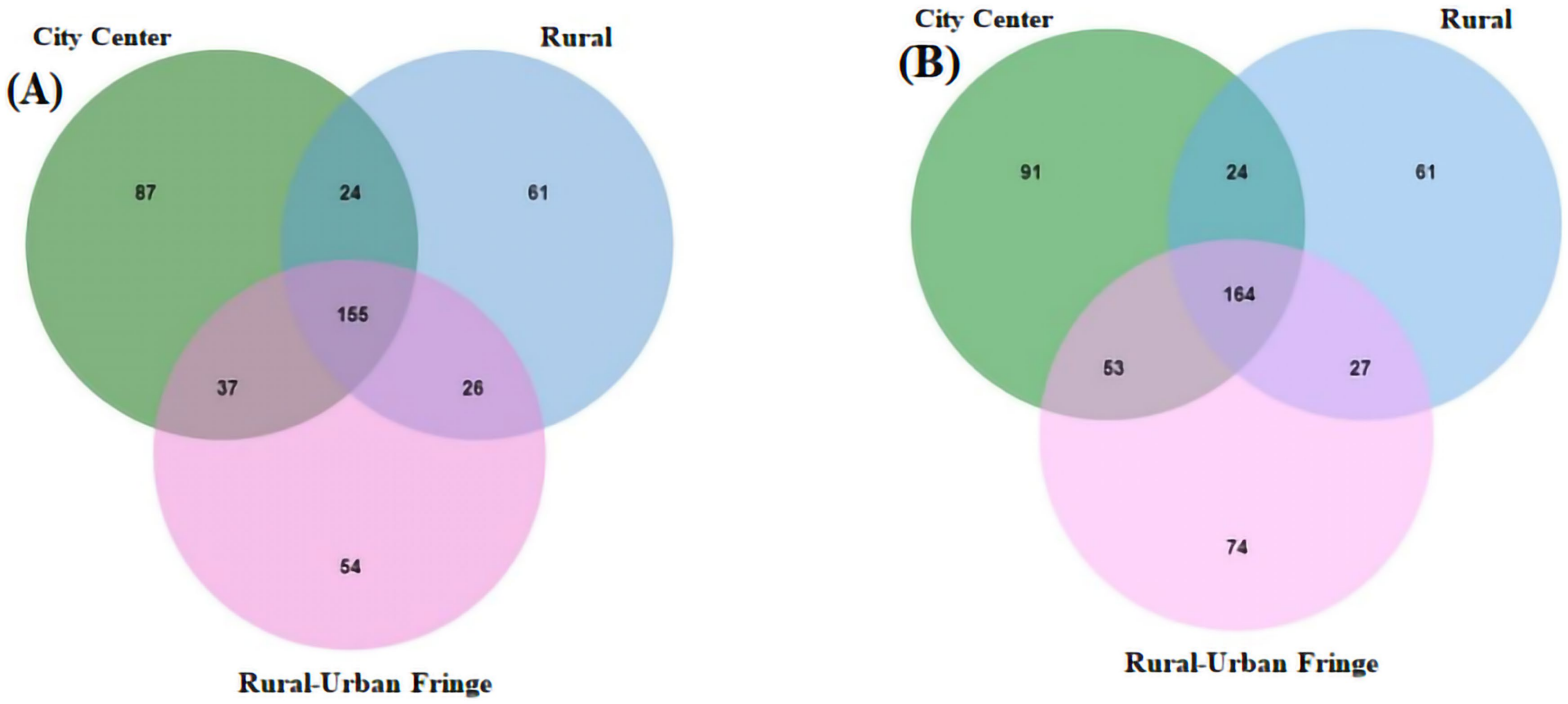
Area interaction Venn diagram of virus community structure levels. **(A)** Genus level; **(B)** Species level.

The regional interaction Venn diagram of the viral communities in PM_2.5_ at the genus level is shown in Figure 4, and the results of the cross-tabulation analysis indicate that the viral genera shared by the regions are significantly differentiated at the species level, showing obvious region-specific distribution characteristics. For example, within the commonly detected *Enterovirus B* genus across all three regions, 14 viruses, including Coxsackieviruses A9, B2, B4, and B6, were endemic to the rural–urban fringe areas, while Echovirus E11 and B80 were found only in the city center areas. The summary of virus species detected exclusively in the rural–urban fringe area are presented in Supplementary Table S3.

In addition, differences at the species level were observed within the same viral genus. For instance, *Beta-papillomavirus 2* was detected in rural–urban fringe areas with Human papillomavirus 107, 120, and 209, whereas in the city center area, there was only Human papillomavirus 17. This further highlights the spatial heterogeneity in virus species distribution.

#### 3.3.3 Regional distribution characteristics of virus classifications

Comparing the distribution of plant virus, human host virus, and other animal virus in city center, rural–urban fringe, and rural areas, Results revealed a consistent trend: the highest abundance was observed in city center areas, followed by rural–urban fringe areas, with the lowest in rural regions. This pattern suggests a potential association between the degree of urbanization and the enrichment of certain virus host types. The chi-square test showed that the difference in the distribution of virus host types among the different regions was statistically significant (*χ*^2^ = 21.098; *p* = 0.002), as detailed in Table 6.

**Table 6:** Regional distribution statistics of virus classifications (%).

Classification	Site			Total	*χ^2^*	*P*
	City center	Rural–urban fringe	Rural			
Plant viruses	40.7	31.0	28.3	100.0	21.098	0.002
Human host viruses	37.2	36.9	25.9	100.0		
Other animal viruses	37.1	34.3	28.6	100.0		
Other viruses	36.3	29.3	34.4	100.0		

#### 3.3.4 Composition of viral community structure

**Figure 5:**
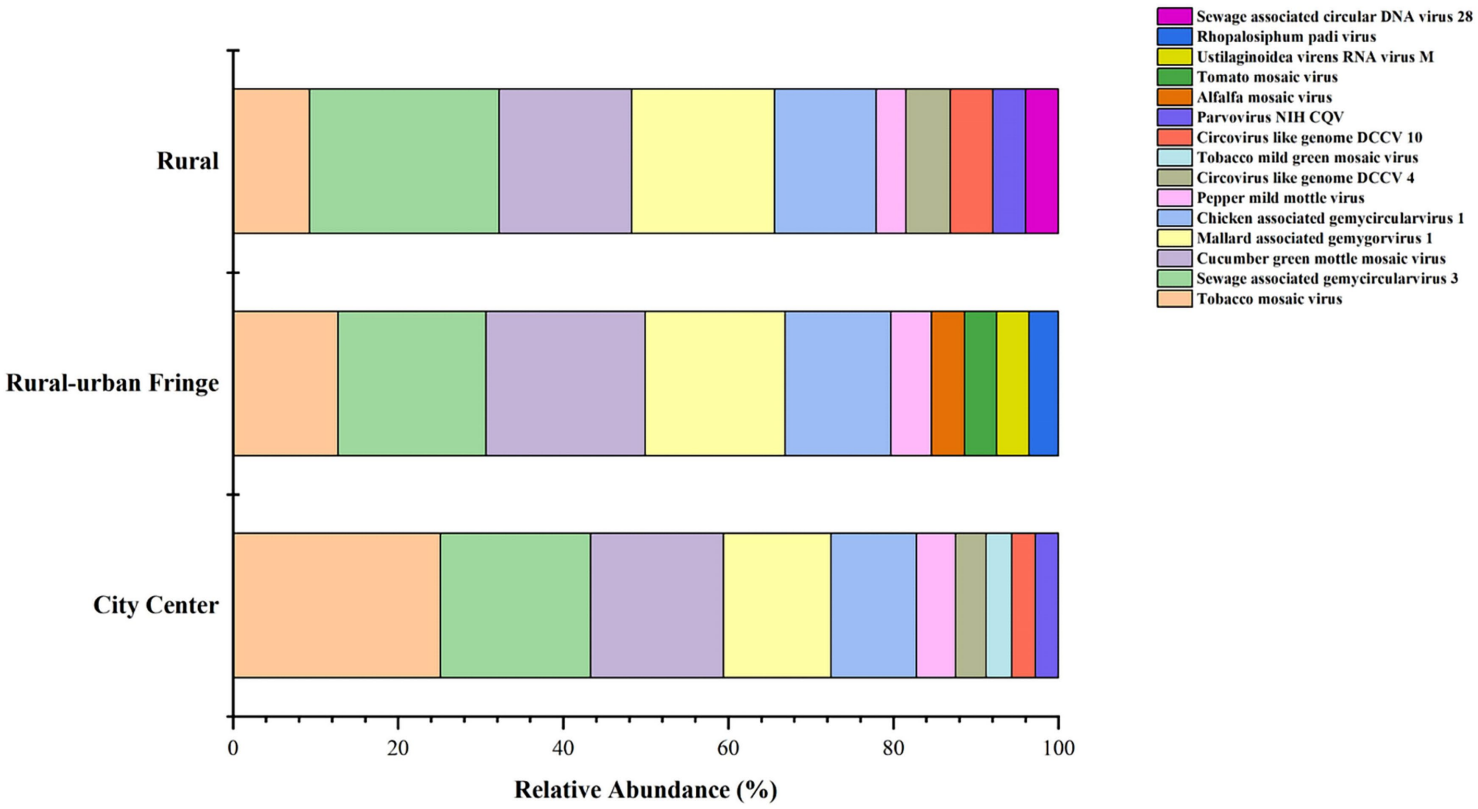
Area distribution of the top 10 most abundant viral species.

A detailed analysis of the relative abundance of viruses across the three regions is shown in Figure 5. Notably, only the rural–urban fringe area exhibited a discrepancy between the dominant viruses at the genus and species levels. In this region, a total of 25 *Enterovirus B* group-related species were detected; however, their relative abundances were generally low.

Further analysis of the top ten viruses by relative abundance in the three regions revealed that six viral types were shared among all regions (Supplementary Table S5). However, the most abundant virus varied across each region.

### 3.4 Spatial and temporal distribution of viruses

In winter, plant viruses, other animal viruses, and other viruses had the highest proportions in the city center area and the lowest in the rural–urban fringe area. In contrast, human host viruses were most prevalent in the rural–urban fringe and least in the rural area (Table 7). Notably, a large number of coxsackieviruses and echoviruses from various subtypes of the *Enterovirus B* genus were detected in the rural–urban fringe area during this period.

**Table 7:** Spatial and temporal distribution of classified viruses.

Classification		City center	Rural–urban fringe	Rural	Total
Winter	Plant viruses	39.8	30.0	30.2	100.0
	Human host viruses	37.1	41.4	21.6	100.0
	Other animal viruses	35.9	31.2	32.9	100.0
	Other viruses	35.2	27.5	37.3	100.0
Spring	Plant viruses	40.6	31.5	27.9	100.0
	Human host viruses	29.3	39.7	31.0	100.0
	Other animal viruses	37.7	36.7	25.6	100.0
	Other viruses	34.3	30.6	35.1	100.0
Summer	Plant viruses	44.5	34.1	21.4	100.0
	Human host viruses	64.1	28.3	7.6	100.0
	Other animal viruses	29.0	37.6	23.4	100.0
	Other viruses	43.8	31.4	24.8	100.0

In spring, the viral distribution patterns shifted. Plant viruses and other animal viruses continued to show higher proportions in the rural–urban fringe area and lower in the rural area. However, human host viruses exhibited a unique pattern, with the highest proportion in the rural–urban fringe area and a moderate level in the rural area, reflecting the impact of seasonal changes on virus distribution in densely populated areas.

By summer, the proportions of plant viruses, human host viruses, and other viruses in the city center area all peaked. During this period, various subtypes of influenza A virus were detected in the city center area. However, their relative abundances were all zero, suggesting the potential risk of influenza A virus transmission in the city, though no effective spread had occurred at that time.

### 3.5 Relationship between human host viruses and air quality and meteorological factors

#### 3.5.1 Data inclusion

The human host viruses included in this study include 2019-nCoV, Human herpesvirus 5, Human herpesvirus 4, Human immunodeficiency virus 1 (HIV-1), and *Mammalian rubulavirus 5*, with sample counts of 58, 24, 23, 17, and 17, respectively. These viruses represent all human-associated viruses detected through metaviromic sequencing, without applying any additional selection criteria. To explore the relationship between viral relative abundance and environmental factors, we divided the relative abundance of each virus into high abundance group (top 50%) and low abundance group (bottom 50%) according to the median, and carried out multivariate regression analysis by combining the air pollutant data and meteorological data.

#### 3.5.2 Logistic regression analysis of 2019-nCoV

The results of the logistic regression analysis for 2019-nCoV are shown in Table 8. The analysis revealed that only SO_2_ in the air quality factor is significantly associated with the relative abundance of 2019-nCoV (*OR* = 0.498; *p* = 0.034). All other air quality and meteorological factors showed no significant association with 2019-nCoV relative abundance (*p* > 0.05), suggesting that higher atmospheric concentrations of SO_2_ may be linked to a decreased relative abundance of 2019-nCoV. It should be noted that the regression analysis in Table 8 has adjusted for seasonal factors and regional differences to control their potential confounding effects.

**Table 8:** Logistic regression analysis of the relative abundance of 2019-nCoV with air quality and meteorological factors.

Variable	Logistic		
	OR	95% CI	*p*
O_3_	0.999	0.974–1.026	0.969
PM_10_	1.099	0.969–1.246	0.140
PM_2.5_	0.976	0.836–1.139	0.755
NO_2_	0.986	0.900–1.079	0.754
SO_2_	0.498	0.261–0.947	0.034
Average air pressure	1.017	0.944–1.096	0.655
Average temperature	0.974	0.904–1.049	0.491
Average relative humidity	0.981	0.828–1.161	0.819
Daily average wind speed	0.715	0.131–3.915	0.699
Sunshine hours	1.018	0.754–1.373	0.910

#### 3.5.3 Results of logistic regression analysis of human herpesvirus 4 and 5, HIV-1, and *mammalian rubulavirus 5*

The logistic regression analysis of the relative abundance of human herpesvirus 5 (see Table 9) indicated no significant associations between this virus and any air quality or meteorological factors (*p* > 0.05). However, a potential association was found with daily average wind speed (*OR* = 0.002; *p* = 0.045), suggesting that an increase in average daily wind speed may be linked to a decrease in the relative abundance of human herpesvirus 5. It should be noted that the regression analysis in Table 9 has adjusted for seasonal factors and regional differences to control their potential confounding effects.

**Table 9:** Logistic regression analysis of the relative abundance of human herpesvirus 5 with air quality and meteorological factors.

Variable	Logistic		
	OR	95% CI	*p*
O_3_	1.003	0.957–1.051	0.902
PM_10_	0.930	0.736–1.175	0.544
PM_2.5_	1.298	0.922–1.827	0.134
NO_2_	1.013	0.832–1.232	0.901
SO_2_	1.243	0.438–3.532	0.683
Average air pressure	0.967	0.916–1.020	0.220
Average temperature	0.853	0.629–1.156	0.305
Average relative humidity	0.848	0.672–1.071	0.166
Daily average wind speed	0.002	0.000–0.877	0.045
Sunshine hours	1.143	0.544–2.399	0.724

As for human herpesvirus 4, HIV-1, and *Mammalian rubulavirus 5*, logistic regression analysis showed no significant associations between their relative abundance and air quality or meteorological factors (*p* > 0.05).

## 4 Discussion

### 4.1 Seasonal distribution characteristics of virus levels carried by PM_2.5_

In Fuzhou, the climate during spring and summer is characterized by high temperatures and humidity, while winter features alternating dry and wet conditions. These seasonal differences may impose varying selective pressures on microbial survival (13), which in turn can affect their transmission and dispersion in the atmosphere (14). It has been shown that the number of *Streptococcus pneumoniae* in the atmosphere peaks in the winter and decreases significantly in the summer (15). In addition, the transmission of many viruses exhibits clear seasonal patterns, which are closely associated with local climatic factors (16). For instance, a study conducted at a nursery school in Virginia, USA, found that both the abundance and diversity of RNA viruses were lower during the hot and humid summer than in winter, indicating a pronounced seasonal differentiation (17). A study in Italy also revealed significant seasonal differences in COVID-19 mortality rates, further supporting the role of climatic factors in modulating viral activity cycles (18).

The findings of this study are consistent with those of previous reports. However, in this study, the difference in RNA virus proportions between winter and summer was relatively small, and the proportion in spring was even lower than in summer. This may be attributed to the detection of a large number of influenza A viruses in the summer samples, despite the relative abundance of each subtype being zero. As a result, when viruses were classified by host type and their seasonal shifts were analyzed from spring to summer, the decrease in plant viruses and other animal viruses was more pronounced than that of human pathogenic viruses.

Seasonal differences were also observed in the relative abundance of dominant viruses, which may be attributed to two main factors. First, the concentration of aerosols in the atmosphere can influence the relative abundance of viruses (19). Previous studies have shown that the spread of SARS-CoV-2 is significantly positively correlated with meteorological factors such as PM_2.5_, CO, and NO₂ (20, 21). Moreover, aerosols may facilitate the long-term spread of the virus in the global atmosphere (8). A study in Italy indicated that elevated PM_2.5_ levels may facilitate the spread of COVID-19, suggesting that air pollutants could indirectly influence the intensity of viral transmission among human populations (22). Second, Fuzhou experiences intense sunlight during the summer, and strong solar radiation may contribute to virus inactivation under high-light conditions (23).

In addition, human pathogenic viruses were detected among the dominant viral genera in both spring and summer, specifically *Parvovirus* NIH-CQV and human adenovirus group C, respectively. *Parvovirus* NIH-CQV was first isolated in 2013 from the serum of a patient with hepatitis by a joint Chinese-American research team (24). Subsequent studies suggested that it might be a laboratory contaminant, potentially originating from centrifuge columns contaminated by marine viruses (25). Since then, there have been few reports on this virus, and the reason for its detection in the current sequencing analysis warrants further investigation.

### 4.2 Distribution characteristics of viral levels carried by PM_2.5_ in different regions

In this study, the average number of viral genera and species in PM_2.5_ samples showed an increasing trend from rural to rural–urban fringe to city center areas. Further compositional analysis of plant viruses, human host viruses, and other animal viruses across the city center, rural–urban fringe, and rural regions revealed that all three types had the highest proportions in the city center area, indicating a clear urban gradient in viral distribution.

This distribution pattern may be influenced by multiple factors, including the level of regional urbanization, dominant economic industries, and human activities in each area. In northeastern China, for instance, provinces and cities with heavy industry as their main economic sector have been found to host a higher abundance of airborne microorganisms compared to coastal cities focused on high-tech industries (26, 27). Furthermore, regional differences in viral relative abundance have been observed between inland and coastal cities (28). A 2023 study found that, although various functional zones in several European cities differed greatly in terms of human activities and pollution sources, no significant differences were detected in viral evenness or diversity indices (29), which fits with the results of this study. The reasons for this may be twofold: first, Fuzhou has been actively implementing air quality improvement initiatives in recent years, potentially reducing viral diversity across its economic zones; second, the city’s large green space coverage may help suppress viral dispersion (30). Therefore, refined air quality management and health risk assessment should be strengthened for more urbanized areas with higher viral abundance.

### 4.3 Influencing factors of human host viruses

Logistic regression analysis in this study revealed that SO_2_ may have an inhibitory effect on the relative abundance of 2019-nCoV. Although SO_2_ is a common air pollutant known to pose health risks to humans, it also possesses antimicrobial and antioxidant properties and is therefore widely used in food preservation (31). Some studies have shown that low-level environmental exposure to SO₂ may have an acute protective effect against bacterial lung infections and can even reduce outpatient visits for tuberculosis patients (32), suggesting that this gaseous compound may have certain biological regulatory functions within a specific concentration range.

Among the meteorological factors, the study also found a significant negative correlation between daily average wind speed and the relative abundance of human herpesvirus 5. Previous research has indicated that COVID-19 can spread over longer distances under higher wind speed conditions, likely due to enhanced air mobility (33). Additionally, A study on the correlation between meteorological factors and the number of COVID-19 cases in Turkey showed that the strongest correlation between wind speed and the total number of cases was found to have a lag effect (34), confirming that wind speed accelerates air movement, resulting in the spread of the virus. In light of the above findings, it is recommended that in areas with high PM2.5 concentrations and viral loads, intervention measures such as strengthening air quality monitoring and improving ventilation in public places be implemented to reduce transmission risks.

This study utilizes metaviromic techniques to reveal the diversity of viruses in atmospheric PM2.5 and their influencing factors. It is representative of cross-seasonal and multi-regional samples, expanding the perspective of environmental virology research. However, the samples were collected over a short time span, and the study is an ecological observational analysis, making it difficult to establish a causal relationship between virus distribution and climatic factors. This limitation requires further investigation in the future through a combination of experimental studies and long-term monitoring.

## 5 Conclusion

Although urban air quality has improved in recent years, metaviromic analysis of PM_2.5_ samples from Fuzhou revealed the continued presence of diverse viral communities in the atmosphere, with distinct seasonal and regional patterns, indicating that viral exposure in urban environments remains a concern. The study found that viral diversity exhibits significant seasonal variations, with winter showing significantly higher levels than other seasons. Additionally, SO_2_ concentration and wind speed were negatively correlated with the abundance of some human viruses, suggesting that air pollutants and meteorological conditions may influence viral distribution and transmission. Therefore, this study utilized metaviromics to reveal the temporal–spatial characteristics of viral communities in PM_2.5_ and their environmental influencing factors, providing data support for understanding the potential health risks of airborne viruses and offering a scientific basis for urban air pollution control strategies and viral transmission risk assessment.

## Data Availability

The data analyzed in this study is subject to the following licenses/restrictions: The data are not publicly available due to privacy. Requests to access these datasets should be directed to: wcc@fjmu.edu.cn.

## Footnotes

^1^ https://www.ncbi.nlm.nih.gov/
