# Supplementary material for: Analysis of the distribution characteristics and influencing factors of viruses carried by atmospheric PM2.5 based on metaviromics
Source: Front Public Health. 2025 Jun 25;13:1616737. doi: 10.3389/fpubh.2025.1616737 (PMC12237916; doi:10.3389/fpubh.2025.1616737)
Supplement: Supplementary file 1 [file Data_Sheet_1.docx]

Supplementary Material

# Supplementary Figures and Tables

## Supplementary Figures

**Supplementary Figure S1.** Geographical distribution of PM_2.5_ sampling sites in Fuzhou. A: City Center; B: Rural-Urban Fringe; C: Rural


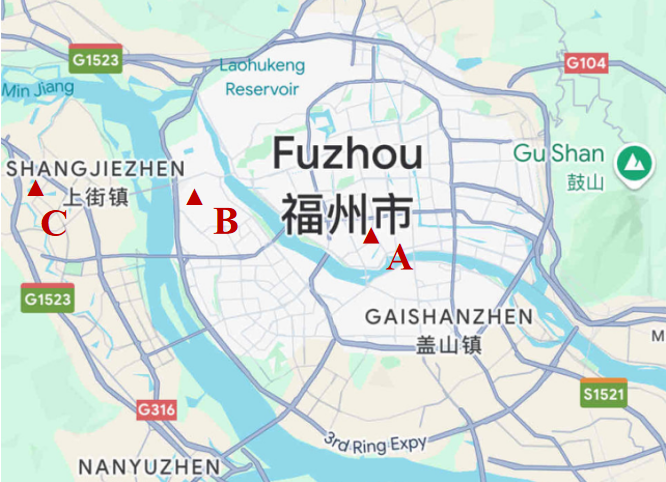


**Supplementary Figure S2.** Field deployment configuration of MH1205 samplers ( Hongtang Central Primary School)


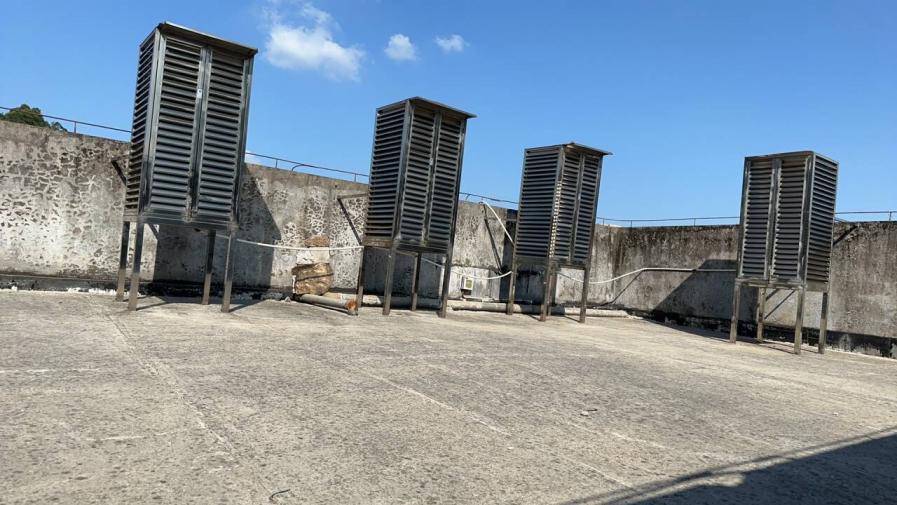


**Supplementary Figure S3.** Basic principle of the MH1205 constant temperature and constant flow air/particulate sampler


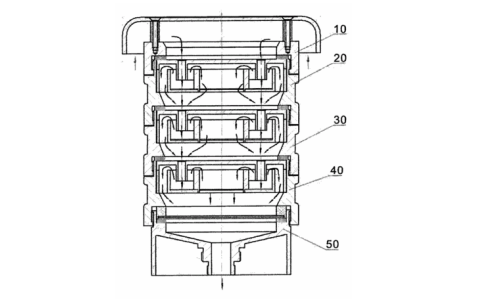


## Supplementary Tables

**Supplementary Table S1.** Distribution of weight differences before and after sampling of quartz filter membranes for PM_2.5_ samples by sampling site and season (Mean and Standard Deviation)

| Site | Season | Weight (g)  Mean ± SD |
| --- | --- | --- |
| City Center | spring | 5.86±2.23 |
|  | summer | 3.37±1.83 |
|  | winter | 9.35±6.19 |
| Rural-urban Fringe | spring | 5.23±2.62 |
|  | summer | 3.23±1.31 |
|  | winter | 8.39±4.42 |
| Rural | spring | 4.30±1.76 |
|  | summer | 2.55±1.19 |
|  | winter | 7.53±4.33 |

**Supplementary Table S2.** Summary of virus species detected exclusively in spring

| Virus Species | Classification | |
| --- | --- | --- |
| Ageratum latent virus | Plant Virus | DNA |
| Blackberry chlorotic ringspot virus | Plant Virus | DNA |
| Carnation Italian ringspot virus | Plant Virus | DNA |
| Chilli veinal mottle virus | Plant Virus | DNA |
| Cucumber necrosis virus | Plant Virus | DNA |
| Cymbidium ringspot virus | Plant Virus | DNA |
| Dill cryptic virus 2 | Plant Virus | DNA |
| Maize necrotic streak virus | Plant Virus | DNA |
| Moroccan pepper virus | Plant Virus | DNA |
| Narcissus symptomless virus | Plant Virus | DNA |
| Panicum mosaic virus | Plant Virus | DNA |
| Parietaria mottle virus | Plant Virus | DNA |
| Pelargonium necrotic spot virus | Plant Virus | DNA |
| Thin paspalum asymptomatic virus | Plant Virus | DNA |
| Tomato bushy stunt virus | Plant Virus | DNA |
| Grapevine Algerian latent virus | Plant Virus | RNA |
| Human associated gemykibivirus 5 | Human Host Virus | DNA |
| Human papillomavirus 17 | Human Host Virus | DNA |
| Human polyomavirus 5 | Human Host Virus | DNA |
| Sewage derived gemygorvirus 1 | Human Host Virus | DNA |
| Enterovirus D68 | Human Host Virus | RNA |
| Influenza A virus H3N2 | Human Host Virus | RNA |
| Influenza A virus H4N6 | Human Host Virus | RNA |
| Influenza A virus H5N2 | Human Host Virus | RNA |
| Influenza A virus H5N3 | Human Host Virus | RNA |
| Aedes albopictus densovirus 2 | Other Animal Virus | DNA |
| Acanthamoeba polyphaga mimivirus | Other Animal Virus | DNA |
| Blackbird associated gemykibivirus 1 | Other Animal Virus | DNA |
| Choristoneura murinana nucleopolyhedrovirus | Other Animal Virus | DNA |
| Diadromus pulchellus ascovirus 4a | Other Animal Virus | DNA |
| Gerygone associated gemycircularvirus 1 | Other Animal Virus | DNA |
| Llama faeces associated circular DNA virus 1 | Other Animal Virus | DNA |
| Murine astrovirus | Other Animal Virus | DNA |
| Pandoravirus inopinatum | Other Animal Virus | DNA |
| Rat associated gemycircularvirus 1 | Other Animal Virus | DNA |
| Trichoplusia ni single nucleopolyhedrovirus | Other Animal Virus | DNA |
| White spot syndrome virus | Other Animal Virus | DNA |
| Avian avulavirus 6 | Other Animal Virus | RNA |
| Beihai tombus like virus 13 | Other Animal Virus | RNA |
| Changjiang narna like virus 3 | Other Animal Virus | RNA |
| Bovine viral diarrhea virus 1 | Other Animal Virus | RNA |
| Canine vesivirus | Other Animal Virus | RNA |
| Changjiang crawfish virus 3 | Other Animal Virus | RNA |
| Changjiang tombus like virus 12 | Other Animal Virus | RNA |
| Culex flavivirus | Other Animal Virus | RNA |
| Feline astrovirus 2 | Other Animal Virus | RNA |
| Hubei odonate virus 1 | Other Animal Virus | RNA |
| Hubei picorna like virus 79 | Other Animal Virus | RNA |
| Hubei tetragnatha maxillosa virus 5 | Other Animal Virus | RNA |
| Hubei tombus like virus 28 | Other Animal Virus | RNA |
| Hubei Wuhan insect virus 9 | Other Animal Virus | RNA |
| Lake Sinai virus | Other Animal Virus | RNA |
| Rabbit coronavirus HKU14 | Other Animal Virus | RNA |
| Shahe isopoda virus 1 | Other Animal Virus | RNA |
| Shahe picorna like virus 5 | Other Animal Virus | RNA |
| Wenzhou channeled applesnail virus 1 | Other Animal Virus | RNA |
| Wenzhou shrimp virus 8 | Other Animal Virus | RNA |
| Wuhan aphid virus 1 | Other Animal Virus | RNA |
| Caribou associated gemykrogvirus 1 | Other Virus | DNA |
| Sewage associated gemycircularvirus 7 | Other Virus | DNA |
| Sewage associated gemycircularvirus 9 | Other Virus | DNA |

**Supplementary Table S3.** Summary of virus species detected exclusively in the rural-urban fringe area

| Virus Species | Classification | |
| --- | --- | --- |
| Ageratum latent virus | Plant Virus | RNA |
| Blackberry chlorotic ringspot virus | Plant Virus | RNA |
| Carnation mottle virus | Plant Virus | RNA |
| Carrot mottle virus | Plant Virus | RNA |
| Chilli veinal mottle virus | Plant Virus | RNA |
| Cymbidium mosaic virus | Plant Virus | RNA |
| Dill cryptic virus 2 | Plant Virus | RNA |
| Hydrangea ringspot virus | Plant Virus | RNA |
| Narcissus symptomless virus | Plant Virus | RNA |
| Parietaria mottle virus | Plant Virus | RNA |
| Watermelon silver mottle tospovirus | Plant Virus | RNA |
| Wild tomato mosaic virus | Plant Virus | RNA |
| Adeno associated virus 7 | Human Host Virus | DNA |
| Human papillomavirus 105 | Human Host Virus | DNA |
| Human papillomavirus 107 | Human Host Virus | DNA |
| Human papillomavirus 120 | Human Host Virus | DNA |
| Human papillomavirus type 16 | Human Host Virus | DNA |
| Human papillomavirus type 209 | Human Host Virus | DNA |
| Sewage derived gemygorvirus 1 | Human Host Virus | DNA |
| Torque teno virus | Human Host Virus | DNA |
| Breda virus | Human Host Virus | RNA |
| Coxsackievirus A12 | Human Host Virus | RNA |
| Coxsackievirus A7 | Human Host Virus | RNA |
| Coxsackievirus A9 | Human Host Virus | RNA |
| Coxsackievirus B2 | Human Host Virus | RNA |
| Coxsackievirus B4 | Human Host Virus | RNA |
| Coxsackievirus B6 | Human Host Virus | RNA |
| Echovirus E15 | Human Host Virus | RNA |
| Echovirus E18 | Human Host Virus | RNA |
| Echovirus E19 | Human Host Virus | RNA |
| Echovirus E3 | Human Host Virus | RNA |
| Echovirus E9 | Human Host Virus | RNA |
| Enterovirus B77 | Human Host Virus | RNA |
| Enterovirus B81 | Human Host Virus | RNA |
| Enterovirus B97 | Human Host Virus | RNA |
| Human enterovirus 107 | Human Host Virus | RNA |
| Swine vesicular disease virus | Human Host Virus | RNA |
| Adelie penguin polyomavirus | Other Animal Virus | DNA |
| African swine fever virus | Other Animal Virus | DNA |
| Apis mellifera filamentous virus | Other Animal Virus | DNA |
| Chicken stool associated gemycircularvirus | Other Animal Virus | DNA |
| Choristoneura murinana nucleopolyhedrovirus | Other Animal Virus | DNA |
| Diadromus pulchellus ascovirus 4a | Other Animal Virus | DNA |
| Duck faeces associated circular DNA virus 1 | Other Animal Virus | DNA |
| Equine associated gemycircularvirus 1 | Other Animal Virus | DNA |
| Faeces associated gemycircularvirus 1 | Other Animal Virus | DNA |
| Gerygone associated gemycircularvirus 1 | Other Animal Virus | DNA |
| Mongoose associated gemykibivirus 1 | Other Animal Virus | DNA |
| Orgyia pseudotsugata multiple  nucleopolyhedrovirus | Other Animal Virus | DNA |
| Pygoscelis adeliae polyomavirus 1 | Other Animal Virus | DNA |
| Avian avulavirus 6 | Other Animal Virus | RNA |
| Boolarra virus | Other Animal Virus | RNA |
| Bovine viral diarrhea virus 1 | Other Animal Virus | RNA |
| Changjiang tombus like virus 7 | Other Animal Virus | RNA |
| Dromedary astrovirus | Other Animal Virus | RNA |
| Hubei leech virus 2 | Other Animal Virus | RNA |
| Hubei odonate virus 1 | Other Animal Virus | RNA |
| Hubei picorna like virus 79 | Other Animal Virus | RNA |
| Hubei tetragnatha maxillosa virus 5 | Other Animal Virus | RNA |
| Hubei tombus like virus 28 | Other Animal Virus | RNA |
| Hubei tombus like virus 5 | Other Animal Virus | RNA |
| Israeli acute paralysis virus | Other Animal Virus | RNA |
| Picornavirales Bu 3 | Other Animal Virus | RNA |
| Porcine sapelovirus 1 | Other Animal Virus | RNA |
| Rabbit coronavirus HKU14 | Other Animal Virus | RNA |
| Santeuil nodavirus | Other Animal Virus | RNA |
| Sanxia atyid shrimp virus 2 | Other Animal Virus | RNA |
| Shahe isopoda virus 1 | Other Animal Virus | RNA |
| Siniperca chuatsi rhabdovirus | Other Animal Virus | RNA |
| Wenzhou channeled applesnail virus 1 | Other Animal Virus | RNA |
| Caribou associated gemykrogvirus 1 | Other Virus | DNA |
| Sewage associated circular DNA virus 15 | Other Virus | DNA |
| Fusarium graminearum dsRNA mycovirus 4 | Other Virus | RNA |

**Supplementary Table S4.** Top 10 most abundant viral species detected in PM_2.5_ by season with their relative abundances and virus classification

| Season | Rank | Detected Virus Species | Relative Abundance | Relative Abundance (%) | Virus Classification |
| --- | --- | --- | --- | --- | --- |
| Winter | 1 | Tobacco mosaic virus | 722.12 | 24.52 | Plant Virus |
|  | 2 | Sewage associated gemycircularvirus 3 | 468.61 | 15.91 | Other Animal Virus |
|  | 3 | Cucumber green mottle mosaic virus | 381.91 | 12.97 | Plant Virus |
|  | 4 | Mallard associated gemygorvirus 1 | 325.17 | 11.04 | Other Animal Virus |
|  | 5 | Chicken associated gemycircularvirus 1 | 253.14 | 8.60 | Other Animal Virus |
|  | 6 | Pepper mild mottle virus | 194.64 | 6.61 | Plant Virus |
|  | 7 | Circovirus like genome DCCV 4 | 190.88 | 6.48 | Other Virus |
|  | 8 | Alfalfa mosaic virus | 162.65 | 5.52 | Plant Virus |
|  | 9 | Tomato mosaic virus | 130.34 | 4.43 | Plant Virus |
|  | 10 | Tobacco mild green mosaic virus | 115.27 | 3.91 | Plant Virus |
| Spring | 1 | Sewage associated gemycircularvirus 3 | 601.93 | 22.70 | Other Animal Virus |
|  | 2 | Mallard associated gemygorvirus 1 | 509.20 | 19.21 | Other Animal Virus |
|  | 3 | Cucumber green mottle mosaic virus | 431.64 | 16.28 | Plant Virus |
|  | 4 | Chicken associated gemycircularvirus 1 | 367.57 | 13.86 | Other Animal Virus |
|  | 5 | Tobacco mosaic virus | 293.38 | 11.07 | Plant Virus |
|  | 6 | Parvovirus NIH CQV | 119.71 | 4.52 | Human Host Virus |
|  | 7 | Sewage associated circular DNA virus 28 | 89.36 | 3.37 | Other Virus |
|  | 8 | Circovirus like genome DCCV 10 | 86.62 | 3.27 | Other Virus |
|  | 9 | Pepper mild mottle virus | 80.93 | 3.05 | Plant Virus |
|  | 10 | Circovirus like genome DCCV 4 | 70.82 | 2.67 | Other Virus |
| Summer | 1 | Cucumber green mottle mosaic virus | 358.89 | 24.06 | Plant Virus |
|  | 2 | Sewage associated gemycircularvirus 3 | 290.71 | 19.49 | Other Animal Virus |
|  | 3 | Mallard associated gemygorvirus 1 | 245.25 | 16.44 | Other Animal Virus |
|  | 4 | Chicken associated gemycircularvirus 1 | 190.33 | 12.76 | Other Animal Virus |
|  | 5 | Tobacco mosaic virus | 102.27 | 6.86 | Plant Virus |
|  | 6 | Circovirus like genome DCCV 10 | 89.01 | 5.97 | Other Virus |
|  | 7 | Rhopalosiphum padi virus | 74.81 | 5.01 | Other Animal Virus |
|  | 8 | Chicken associated gemycircularvirus 2 | 48.29 | 3.24 | Other Animal Virus |
|  | 9 | Ustilaginoidea virens RNA virus L | 46.40 | 3.11 | Other Virus |
|  | 10 | Cladosporium cladosporioides virus 1 | 45.45 | 3.05 | Other Virus |

**Supplementary Table S5.** Top 10 most abundant viral species detected in PM_2.5_ by site with their relative abundances and virus classification

| Site | Rank | Detected Virus Species | Relative Abundance | Relative Abundance (%) | Virus Classification |
| --- | --- | --- | --- | --- | --- |
| City Center | 1 | Tobacco mosaic virus | 635.67 | 25.26 | Plant Virus |
|  | 2 | Sewage associated gemycircularvirus 3 | 456.50 | 18.14 | Other Animal Virus |
|  | 3 | Cucumber green mottle mosaic virus | 406.19 | 16.14 | Plant Virus |
|  | 4 | Mallard associated gemygorvirus 1 | 327.65 | 13.02 | Other Animal Virus |
|  | 5 | Chicken associated gemycircularvirus 1 | 260.16 | 10.34 | Other Animal Virus |
|  | 6 | Pepper mild mottle virus | 120.26 | 4.78 | Plant Virus |
|  | 7 | Circovirus like genome DCCV 4 | 88.36 | 3.51 | Other Virus |
|  | 8 | Tobacco mild green mosaic virus | 79.77 | 3.17 | Plant Virus |
|  | 9 | Circovirus like genome DCCV 10 | 72.40 | 2.88 | Other Virus |
|  | 10 | Parvovirus NIH CQV | 69.95 | 2.77 | Human Host Virus |
| Rural-urban Fringe | 1 | Cucumber green mottle mosaic virus | 412.63 | 19.43 | Plant Virus |
|  | 2 | Sewage associated gemycircularvirus 3 | 381.53 | 17.97 | Other Animal Virus |
|  | 3 | Mallard associated gemygorvirus 1 | 359.76 | 16.94 | Other Animal Virus |
|  | 4 | Chicken associated gemycircularvirus 1 | 271.63 | 12.79 | Other Animal Virus |
|  | 5 | Tobacco mosaic virus | 268.52 | 12.65 | Plant Virus |
|  | 6 | Pepper mild mottle virus | 105.75 | 4.98 | Plant Virus |
|  | 7 | Alfalfa mosaic virus | 85.01 | 4.00 | Plant Virus |
|  | 8 | Tomato mosaic virus | 83.98 | 3.96 | Plant Virus |
|  | 9 | Ustilaginoidea virens RNA virus M | 80.87 | 3.81 | Other Virus |
|  | 10 | Rhopalosiphum padi virus | 73.61 | 3.47 | Other Animal Virus |
| Rural | 1 | Sewage associated gemycircularvirus 3 | 526.69 | 22.98 | Other Animal Virus |
|  | 2 | Mallard associated gemygorvirus 1 | 397.25 | 17.33 | Other Animal Virus |
|  | 3 | Cucumber green mottle mosaic virus | 367.12 | 16.02 | Plant Virus |
|  | 4 | Chicken associated gemycircularvirus 1 | 283.43 | 12.37 | Other Animal Virus |
|  | 5 | Tobacco mosaic virus | 213.13 | 9.30 | Plant Virus |
|  | 6 | Circovirus like genome DCCV 4 | 121.63 | 5.31 | Other Virus |
|  | 7 | Circovirus like genome DCCV 10 | 116.05 | 5.06 | Other Virus |
|  | 8 | Parvovirus NIH CQV | 91.50 | 3.99 | Human Host Virus |
|  | 9 | Sewage associated circular DNA virus 28 | 90.39 | 3.94 | Other Virus |
|  | 10 | Pepper mild mottle virus | 84.81 | 3.70 | Plant Virus |
